# 

**Published:** 1900-03-15

**Authors:** 


### ANNOUNCEMENTS.

### PENNSYLVANIA BOARD OF DENTAL EXAMINERS.

The Board of Dental Examiners of the State of Pennsylvania will hold examinations simultaneously in Philadelphia and Pittsburg, May 8th, 9th and 10th, and in Philadelphia, June 19th, 20th and 21st. Application for examination must be made to Hon. James W. Latta, Secretary of the Dental Council, Harrisburg, Pa.

G. W. Klump, *Sec'y*,

Williamsport, Pa.

### MEETING OF THE OHIO STATE BOARD OF DENTAL EXAMINERS.

The next meeting of the Ohio Board will be held at the Chittenden Hotel, Columbus, Ohio, beginning Tuesday, May 29th, 1900. Examination will be both theoretical and practical. Applicants are requested to bring instruments rubber dam, filling materials, etc., necessary for making fillings or doing such other work as may be required. Engines will be supplied by the Board. For further particulars or application for examination, write to

DR. L. P. BETHEL, *Sec'y*,
Kent, Ohio.

### KENTUCKY STATE DENTAL ASSOCIATION.

Attention is called to the change of date of the meeting of the Kentucky State Dental Association. On account of change of meeting of Confederate Association and for the purpose of getting railroad rates, we too have changed our date to May 29th, 30th and 31st. We have some thirty papers promised for the meeting and nearly as many clinics, and we will add still others to the list. We have men on the program from about twelve States. Don't fail to come and meet with us, and we promise you a fine time.

F. I. GARDNER, D.D.S., *Sec'y*.

### MICHIGAN STATE DENTAL ASSOCIATION.

At a meeting of the Board of Directors in Detroit, March 8th, the place of meeting of this society was changed from Muskegon to Kalamazoo, and the time set for June 11th, 12th and 13th.

### TRANSPORTATION TO THE INTERNATIONAL DENTAL CONGRESS AT PARIS.

This Transportation Committee has completed arrangements with Thomas Cook & Sons, 251 Broadway, New York City, for the best obtainable rates and accommodations. There will be heavy travel abroad this year, and those contemplating the trip should make application very soon to Messrs. Cook & Sons, at above address, for such accommodations as may be needed.

Several routes and times of sailing are offered:

First.—Sail from New York City, by Red Star Line steamer "Friesland," July 18th, to Antwerp, and thence by rail by way of Brussels to Paris. Return to New York by the same route. Fare for the round trip, first-class passage, $157.85. Slower steamers, same line, the fare would be less

Second.—Sail from New York City, by the North German Lloyd steamers "Barbarossa," July 12th, or "Friederich der Grosse," July 19th, to Cherbourg, thence by rail to Paris, and return by the same route. First-class passage and berth, $177.

Third.—Sail from New York, by Hamburg-American Line Steamers "Pennsylvania," July 14th, or "Pretoria," July 21st, to Cherbourg, and thence by rail to Paris, returning by way of Boulogne-sur-mer and same line to New York. First-class passage and berth, $184.25.

Fourth.—Sail from New York, July 7th, by Steamer "Potsdam," or July 14th by "Statendam," or July 28th by "Rotterdam," to Boulogne-sur-mer, thence by rail to Paris, returning by same route. First-class passage and berth, $163.

Lower fares may be had by three or more occupying the same room, or by taking second-class passage or slower steamers of the several lines. Proportionate rates can be had to Southampton or Liverpool. A two weeks' stay in Paris at a first-class hotel, including carriage drives for three days, twenty admission tickets to the Exposition, transfers, etc., can be had for $65.

A tour to Switzerland for one week, including all expenses, can be had for $50.

A week's tour from Paris to Mayence, thence by steamer down the Rhine to Cologne, thence by rail to Amsterdam, The Hague, Rotterdam, Antwerp, Brussels, and by steamer from Antwerp to London, including all expenses, $42.50.

